# Clinical patterns of rhegmatogenous retinal detachment during the first state of emergency for the COVID-19 pandemic in a Tokyo center

**DOI:** 10.1371/journal.pone.0261779

**Published:** 2021-12-31

**Authors:** Toshiaki Hirakata, Tianxiang Huang, Yoshimune Hiratsuka, Shutaro Yamamoto, Akira Inoue, Akira Murakami

**Affiliations:** Department of Ophthalmology, Juntendo University Faculty of Medicine, Tokyo, Japan; Medizinische Universitat Graz, AUSTRIA

## Abstract

The coronavirus disease 2019 (COVID-19) pandemic is disturbing and overwhelming a regular medical care in the world. We evaluated the clinical characteristics of patients with primary rhegmatogenous retinal detachment (RRD) during the state of emergency for COVID-19 pandemic in Japan. We also reviewed measures against the COVID-19 pandemic in our institute with a focus on RRD treatment. Retrospectively, patients who underwent initial RRD surgery during the state of emergency between April 7, 2020 and May 25, 2020 were included. For comparison, we recruited patients who underwent surgery for initial RRD during the same period in the last 2 years (2018 and 2019). Data related to the number of surgeries, age, gender, macular detachment, proliferative vitreoretinopathy (PVR), preoperative visual acuity, surgical techniques, the time between the onset and hospitalization and/or surgery of the 2020 cohort were analyzed and compared with those of the 2018 and 2019 cohorts. Furthermore, we reviewed measures taken against COVID-19 in our institute. The number of RRD patients during the state of emergency tended to be lower than that within the last 2 years. Relatively lesser female (vs. male) patients were observed in the 2020 cohort than in the last 2 years (*P* = 0.084). In contrast, among all cohorts, no significant differences were observed in the incidence of macula-off and PVR, preoperative visual acuity, and the time period between symptom onset and hospitalization and/or surgery. This is the first report to show the clinical patterns of RRD during COVID-19 pandemic in Japan. Despite the state of emergency for the COVID-19 pandemic, no delay in the patient’s initial visit to the hospital and surgery was observed. Further studies, including multicenter researches, are important for investigating the influence of COVID-19 on urgent ocular diseases.

## Introduction

The current outbreak of the novel severe acute respiratory syndrome coronavirus 2 (SARS-CoV-2) (previously known as 2019-nCoV) was first identified in December 2019 in China. Since then, the entire world has been affected by the coronavirus disease (COVID-19) pandemic. As there is no effective cure, several countries have introduced restrictive measures to control infection. In Japan, the first case was reported in January 2020, and since then, the spread of the infection has been endless. The Japanese government issued a nationwide state of emergency in response to the rapid spread of COVID-19 [1]. Initially, the state of emergency was announced on April 7, 2020 for seven prefectures, i.e., Tokyo, Kanagawa, Saitama, Chiba, Osaka, Kobe, and Fukuoka, where there the number of infected patients has been increasing. Thereafter, the state of emergency was expanded nationwide beginning from April 16, 2020. Once the number of infected people gradually decreased, the state of emergency was lifted on May 25, 2020.

The Department of Ophthalmology, Faculty of Medicine at Juntendo University is located in the center of Tokyo, Japan. Under the state of emergency, the treatment for relative less urgent diseases, such as senile cataract, epiretinal membrane and oculoplastics, was either stopped or postponed in our hospital. However, treatment for urgent diseases, such as rhegmatogenous retinal detachment (RRD), glaucomatous attack, macular hole, and open globe injuries, was unrestricted.

RRD is urgent ophthalmic condition and may result in significant visual loss, especially caused by both, delay in presentation and delay in surgical repair of macula-off retinal detachments [2, 3]. At the onset of RRD, patients typically present with symptoms such as light flashes, floaters, peripheral visual field loss, and blurred vision [4]. According to reports in other countries, COVID-19 and its associated lockdown have changed clinical patterns of RRD; moreover, an increase in the rate of macular detachment (macula-off) and proliferative vitreoretinopathy (PVR) has been observed [5, 6]. However, there are no reports of clinical presentation of RRD during COVID-19 pandemic in Japan. Therefore, evaluating the influence of COVID-19 pandemic and the state of emergency for RRD treatment in Japan is of paramount importance. Here, we evaluated the clinical characteristics of primary RRD during the state of emergency for the COVID-19 pandemic in our institute, Tokyo, Japan.

## Materials and methods

### Subjects

In this retrospective cohort study, patients who underwent evaluation and repair of acute primary RRD in the Department of Ophthalmology, Juntendo University Hospital, Hongo 3-1-3, Bunkyo-ku, Tokyo, Japan, were investigated; all included patients underwent surgery during the state of emergency between April 7, 2020 and May 25, 2020. For comparison, patients who underwent surgery for initial RRD within the same period in 2018 and 2019 were recruited. This retrospective study was conducted in accordance with the Declaration of Helsinki and was approved by the Institutional Review Board of Juntendo University (16–292). All patients signed informed consent forms prior to the RRD surgery. The individual in this manuscript has given written informed consent (as outlined in PLOS consent form) to publish these case details. The number of surgeries, age, gender, macular detachment (macula-on or macula-off), PVR, preoperative visual acuity, surgical techniques, time between onset and hospitalization, the time between onset and surgery, and the period of hospitalization were analyzed. Onset was defined as the time when the patient first noticed subjective symptoms, such as floaters, visual acuity loss, visual field loss, and flashes. Macular detachment was defined using OCT images. All operation were performed by same three surgeon (T.H., A.I., and S.Y.).

### Statistical analysis

Differences in demographic and clinical characteristics between the groups were compared in the patients in 2020 and patients in 2018 and 2019 using Fisher’s exact probability test for categorical variables and the two-sample t-test for continuous variables. The number of primary RRD patients was compared between two groups using one-sample t-test. The number of surgical techniques in two groups (scleral buckle or pars plana vitrectomy (PPV)) was compared using Fisher’s exact test with Holm adjustment. We set the level of statistical significance at P < 0.05 for a two-sided test. All statistical analyses were performed using Stata version 15 (StataCorp, College Station, TX, USA).

## Results

### Comparison of patient demographics and retinal detachment characteristics between cohorts

Table 1 shows the comparison of patient demographics and characteristics for primary RRD during the state of emergency in 2020 and in the same period in the past 2 years (2018 and 2019). During the state of emergency, between April 7, 2020 and May 25, 2020, 20 patients (20 eyes) underwent initial RRD surgery; 35 patients (35 eyes) and 31 patients (31 eyes) underwent initial RRD surgery in 2018 and 2019, respectively. Primary RRD patients in 2020 tended to be lower than those in 2018 and 2019 (*P* = 0.069). Interestingly, there was a tendency toward a lesser number of female patients in 2020 compared with that in the last 2 years (*P* = 0.084). No significant differences among the 2018, 2019, and 2020 cohorts were observed with respect to the incidence of macular detachment, preoperative visual acuity, surgical techniques (scleral buckle *v*.*s*. pars plana vitrectomy (PPV)), the time period between symptom onset and hospitalization, and the time period between symptom onset and the operation for RRD. PVR was not observed in 2018, 2019, and 2020.

**Table 1:** Patient demographics and retinal detachment characteristics.

	2018 and 2019		2020	p value
	2018	2019		
Number (eye)	31 (31)	35 (35)	20 (20)	0.069
Age (SD)	57.2 ± 14.9		54.1 ± 17.5	0.427
	58.1 ± 13.8	56.4 ± 15.8		
Sex, male (%) / female (%)	46 (69.7) / 20 (30.3)		18 (90.0) / 2 (10.0)	0.084
	21 (67.7) / 10 (32.3)	25 (71.4) / 10 (28.6)		
Macula -on (%) / -off (%)	36 (54.5) / 30 (45.5)		12 (60.0) / 8 (40.0)	0.799
	16 (51.6) / 15 (48.4)	20 (57.1) / 15 (42.9)		
LogMAR VA (SD)	0.6 ± 0.7		0.5 ± 1.0	0.744
	0.4 ± 0.5	0.7 ± 0.9		
Surgical techniques: PPV (%) / Buckle (%)	49 (74.2) / 17 (25.8)		15 (75.0) / 5 (25.0)	>0.999
	21 (67.7) / 10 (32.3)	28 (80.0) / 7 (20.0)		
Time between onset to hospitalization (SD) (day)	10.9 ± 19.2		11.7 ± 9.1	0.865
	8.0 ± 10.6	9.0 ± 8.7		
Time between onset to operation (SD) (day)	12.0 ± 19.2		12.1 ± 17.4	0.932
	9.0 ± 10.7	10.0 ± 8.5		

PPV, pars prana vitrectomy; SD, standard deviation; VA, visual acuity.

*: p < 0.05; **p < 0.001.

## Discussion

Here, we document the influence of COVID-19 on patients with RRD at our institute in Tokyo, Japan. At the time of the state of emergency, the number of RRD patients tended to decrease in comparison to that in the last 2 years. In contrast, no significant differences, among 2018, 2019, and 2020 cohorts, were observed in the incidence of macular detachment, preoperative visual acuity, the time period between onset of symptoms and hospitalization, and the time period between onset of symptoms and the operation for RRD. Thus, these findings suggest that the RRD patients in Japan could go to hospitals as usual, without much delay.

According to Jasani et al., despite a reduction in the RRD incidence, an increment in the incidence of macular detachment and PVR was observed during the pandemic lockdown in the United Kingdom [6]. Similarly, Patel et al. reported significantly fewer macula-on RRD patients but with poorer median visual acuity at presentation in the COVID-19 cohort when compared with the control cohort in America [5]. The state of emergency declared by the Japanese government was not legally binding and there were no penalties for individuals who went out [7]; this could have led to the difference between the present study and previous reports from other countries. Although the Japanese government recommended staying home, there was no restriction on gatherings and public transports were not closed. Therefore, the patients could go to hospital/clinics for medical examination whenever they noticed any subjective acute symptoms. In contrast, lockdown in other countries had stricter restrictions [8, 9], which may have resulted in people not visiting the hospital. Moreover, more COVID-19 patients have been observed in other countries in comparison to Japan. Because of the fear of COVID-19, people in other countries might hesitate in visiting hospitals. Indeed, there is evidence of patients with medical emergencies avoiding the emergency department because of fear of contracting COVID-19 [10].

Additionally, the reduced number of RRD in Japan during the state of emergency could be because of a decrease in human activities. Human movement and activity can trigger and progress RRD [11]. During the state of emergency, most Japanese citizens refrained from going outside [7]; in particular, the female RRD patients were markedly decreased in number during the state of emergency. A similar tendency has never been observed in other countries’ previous reports [5, 6]. Interestingly, according to a report, there was a difference in behavior by sex in Japan during the state of emergency [12]. Although, regarding commuting, there was no difference in sex, women activities for personal reasons, such as shopping, dining, socializing, and entertainment, were lower than that of men [12]. The reduced incidence of RRD in the Japanese woman may be due to self-control and decreased outside activity.

At the time when the first state of emergency was declared, there were insufficient PCR test for detection of SARS-CoV-2 in all hospitalized patients in our hospital; testing was limited to the suspected COVID-19 patients. A wide variation in SARS-CoV-2 phenotypes exists between asymptomatic and symptomatic patients [13, 14]. Moreover, the medicals staff was scared of the unknown infectious disease. Therefore, to protect both patients and medical staff, we took the following measures against COVID-19. First, a clear acrylic shield was attached to the slit lamp to prevent droplet infection. In addition, the medical staff wore a face mask and face shield. Because of lack of N95 masks, medical staff used surgical masks. All patients were instructed to wear a face mask in the consultation room. Continuous air ventilation was ensured during examinations in the consultation and examination rooms. Second, all patients were made to wear a face mask during the surgery. Continuous oxygen was maintained around the mouth (3 L/min) under the surgical drape (S1A–S1D Fig). In addition, peripheral arterial oxygen saturation (SpO_2_) was monitored and recorded during operation (S1E Fig). In all patients, SpO_2_ was maintained over 90%, and none of the patients complained of respiratory distress. Based on previous reports, there is evidence that the face mask can protect from COVID-19 [15, 16]. As a result, no medical staff and/or patients were infected with COVID-19. Our measures for prophylaxis of COVID-19, both during examination and surgery, may be beneficial and practical for the patients and medical staff.

Although this report is a first attempt to demonstrate the influence of COVID-19 on patients with RRD in Japan, the results reflect the situation at a single center. Moreover, the number of subjects included in the study cohort was small. Our institute is located in the center of Tokyo, but there may be regional differences between institutes. In Japan, the number of hospitals and clinics varies greatly depending on the region and prefecture. In particular, there are many hospitals in central of Tokyo and few in provinces. In the future, multiple facilities should be investigated to accurately reflect the situation of RRD treatment, during the COVID-19 pandemic, in Japan. The measures against COVID-19, in various institutes, should be reviewed for designing a safer medical system.

## Conclusions

We documented RRD during the first state of emergency against the COVID-19 pandemic in Juntendo University Hospital. This is the first report to show clinical patterns of RRD during the state of emergency against the COVID-19 pandemic in Japan, and RRD patients in our hospital could visit the hospital as usual during the state of emergency. However, since this current study reflects only one facility, it is necessary to conduct multicenter research in the future in order to investigate the exact current situation in Japan. Presently, the COVID-19 pandemic has not ended worldwide; thus, researching clinical patterns of ophthalmic urgent diseases, including RRD is important for future generations.

## Supporting information

S1 Fig Infection defense measures in the operating room. **(A)** A metal bar with oxygen tube is attached to an operating chair (the orange arrow shows the metal bar). **(B)** Patients wearing a mask during the operation (the black arrow shows a surgical mask). **(C)** Near the face of the patient, the oxygen tube was attached. **(D)** Oxygen circulating under the drape supported the breathing of the patient. **(E)** During the operation, the arterial oxygen saturation was monitored.
(PDF)

S1 Data (XLSX)
