# Supplementary figures and images for: Clinical patterns of rhegmatogenous retinal detachment during the first state of emergency for the COVID-19 pandemic in a Tokyo center
Source: PLoS One. 2021 Dec 31;16(12):e0261779. doi: 10.1371/journal.pone.0261779 (PMC8719721; doi:10.1371/journal.pone.0261779)

Supplementary figure 1

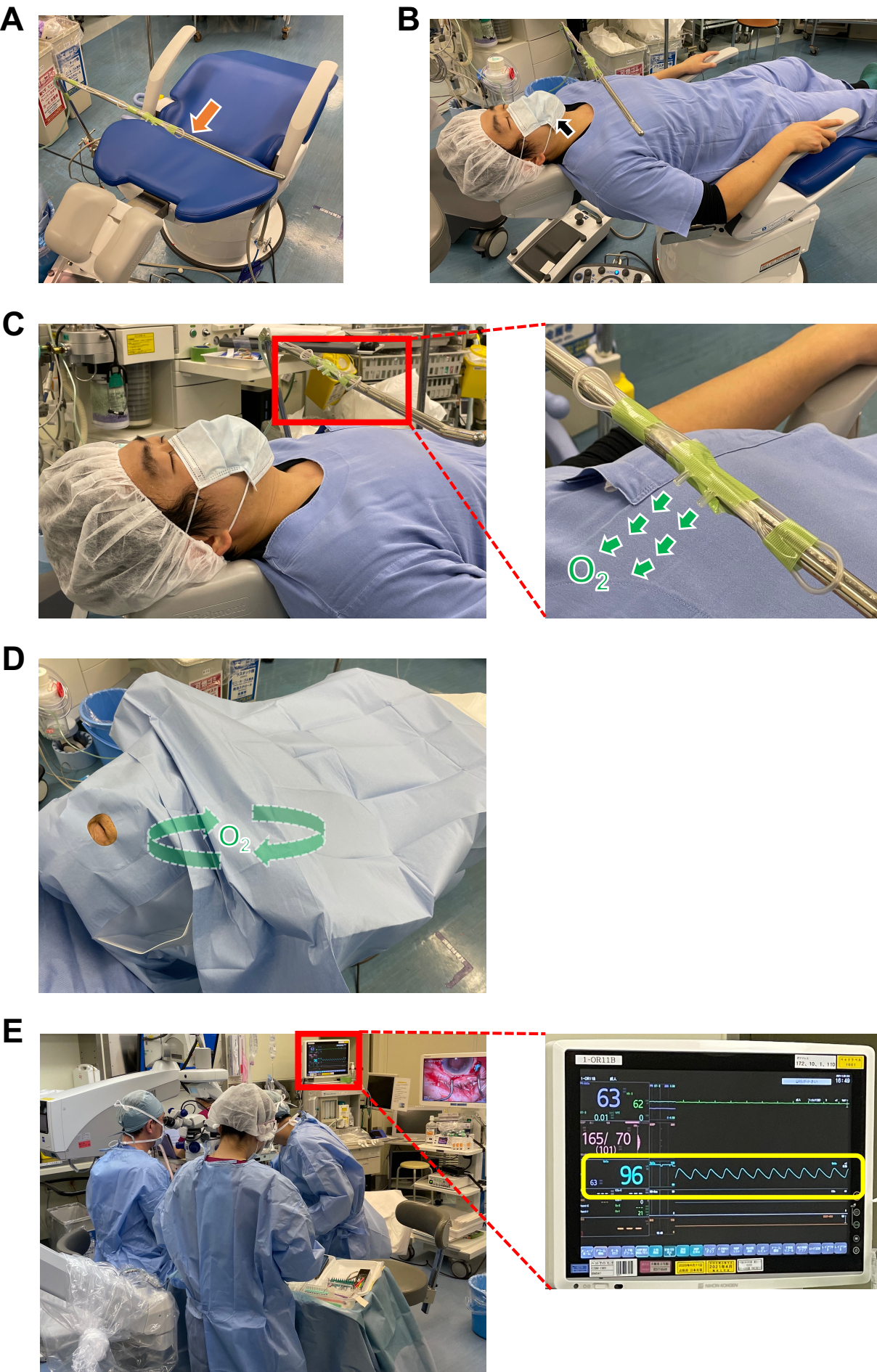

Supplement: S1 Fig — (A) A metal bar with oxygen tube is attached to an operating chair (the orange arrow shows the metal bar). (B) Patients wearing a mask during the operation (the black arrow shows a surgical mask). (C) Near the face of the patient, the oxygen tube was attached. (D) Oxygen circulating under the drape supported the breathing of the patient. (E) During the operation, the arterial oxygen saturation was monitored. (PDF) [file pone.0261779.s001.pdf]
